# Does Insomnia Exist without Hyperarousal? What Else Can There Be?

**DOI:** 10.3390/brainsci10040225

**Published:** 2020-04-10

**Authors:** Célyne Bastien

**Affiliations:** School of psychology, Laval University, Quebec, QC G1V0A6, Canada; celyne.bastien@psy.ulaval.ca

While in ancient Greece, incubation rooms were dedicated to the interpretation of dreams, sleep was also studied by famous philosophers such as Aristotle. In 350 B.C.E., he wrote an essay titled “On Sleep and Sleepiness”. In the essay, he described some basics of how sleep was regulated and how it was naturally induced. Nowadays, we know much more about sleep and its regulation (circadian rhythms, homeostatic processes, etc.) and the diurnal consequences of the lack of sleep (poor memory and decision-making, lack of attention, increased car accidents, health issues, etc.). 

Although some of us might decide today to cut down on our sleep because of work, family or other related reasons, this choice is voluntary. Involuntary sleep reduction, as it is the case with insomnia, brings its own consequences. With the advent of the modern world and the appearance of stress, we have seen cases of insomnia grow from one century to the other. Today, worldwide, rates of chronic insomnia are nearing 12% of the population, while about 30% of individuals report suffering from an occasional bad night of sleep. Although we know that insomnia, albeit acute or chronic, is often precipitated by an event, one can be predisposed to suffer from it (for example, being a woman or an elderly) and can also maintain sleep difficulties with bad habits (for example, staying in bed while unable to fall asleep). 

At the core of insomnia, hyperarousal (cortical and physical) has been recognized as having a dominant role to play. One can be predisposed to have a higher hyperarousal level, or hyperarousal can develop with time. In fact, hyperarousal may be what prevents you from falling asleep and wakes you up during the night, and is reflected through heightened cortical activity, for example. However, hyperarousal might not explain everything. In fact, one perspective offered by Vargas and al. [1] questions if hyperarousal has anything to do with either acute or chronic insomnia. By disentangling how the flight-or-fight response suggested for acute insomnia is different to the learned hyperarousal observed in chronic insomnia, this review sets the tone for the rest of the issue. However, a very innovative way to study arousal, and especially hyperarousal, while looking at socioemotional processing is described by Howlett and colleagues [2]. By studying responses in categorization of face-emotion stimuli and intensity of risk-taking in both good sleepers and individuals suffering of insomnia, these authors suggests that beta activity (which is considered the cortical signature of hyperarousal) interferes differentially in the two studied groups and is not the hallmark of insomnia only. 

Two other reviews in the issue discuss different issues. The first one discusses how important and high the rate of comorbid insomnia and apnea is. According to authors [3], insomnia was long considered secondary to apnea, and it is only recently that clinicians have begun to study how these two sleep disorders can be dealt differently treatment wise and explain why response rate was often so low in both previous sleep disorders in treatment studies. Next, a systematic review informs us about the subtle but very disturbing role insomnia plays in traumatic brain injury patients. It is worth mentioning that insomnia in conjunction with drowsiness will definitely impact on brain injury patients’ daily life [4]. Recent research in the area of traumatic brain injury is so active that another paper from this issue studies this very interesting area of research, especially when we know that both insomnia and drowsiness are often underdiagnosed and thus undertreated in these patients, while remaining persistent complaints [5]. 

One of the aims of the present issue was to demystify some of the less explored concepts, which might, in fact, act as precipitating factors of insomnia, maintain it with an active comorbidity or have health and societal consequences superseding hyperarousal. As such, jumping right in, we know that alcohol interferes with sleep, and its abrupt cessation in regular drinkers can also cause severe insomnia. However, what role does insomnia have in risky behavior when an individual also suffers from insomnia? In fact, student athletes are at a higher risk of driving under the influence of alcohol than non-athlete students and, furthermore, especially if they suffer from insomnia [6].

Finally, the last paper of this issue deals with some physical aspects of our body: choroid, retinal nerve fibers and the inner plexiform layer [7]. Not only were those affected in individuals suffering of insomnia, they were more so as the severity of insomnia increased. This new line of research might pave the way not only for future research on insomnia and tissue degeneration, but also for sleep and its implications together in non-degenerative profiles as measured by optical coherence tomography. 

As more research evolves on insomnia treatment and sleep per se, the future is bright for those working in these areas of research. It is not imminent that insomnia rates will dramatically drop, and more research to understand how it develops and is maintained as well as the best way to treat it are still more than ever needed. Let us disseminate our knowledge, to nurture what will need to be done next.
